# Mental illness and help-seeking behaviours among Middle Eastern cultures: A systematic review and meta-synthesis of qualitative data

**DOI:** 10.1371/journal.pone.0293525

**Published:** 2023-10-26

**Authors:** Farah Elshamy, Ayah Hamadeh, Jo Billings, Aisha Alyafei

**Affiliations:** Division of Psychiatry, University College London, London, United Kingdom; University of Sharjah, UNITED ARAB EMIRATES

## Abstract

**Background:**

Western literature has long explored help-seeking behaviours related to mental health issues. However, this has been relatively neglected in the Middle East despite an increase in mental health needs in the region. The purpose of this review was to conduct a systematic review and qualitative meta-synthesis exploring help-seeking behaviours related to mental health issues in the Middle East.

**Methods:**

We conducted a systematic review and meta-synthesis to gain a comprehensive overview of what is known about mental health and help-seeking behaviours in the Middle East from published qualitative research in the Middle Eastern region. A search of electronic databases (MEDLINE, Embase, CINAHL, PsycINFO and QScience) was carried out from inception to July 2022. The quality of the included studies was assessed using the Critical Appraisal Skills Programme tool, and the review protocol was pre-registered with PROSPERO (Ref: CRD42022311494).

**Results:**

We identified 16 qualitative studies exploring mental health-seeking behaviours in Middle East countries. Facilitators and barriers to help-seeking were captured under six overarching themes. Across all studies, we identified negative attitudes towards seeking help for mental health issues, economic and structural barriers to accessing mental healthcare, and misconceptions surrounding religious beliefs, all of which had a critical role in impacting decisions to seek mental healthcare services. Many sought help from alternative sources, such as traditional healers or family members before consulting a healthcare professional. The role of the family and cultural norms was also identified as key contributors to people’s help-seeking behaviours.

**Conclusions:**

This meta-synthesis indicates the existence of many challenges surrounding mental health-seeking in the Middle East, including public and internalizing stigmas. This suggests an urgent need for an increase in psychoeducation and mental health awareness in the region.

## Introduction

Whereas Western literature has explored the issue of mental health and help-seeking in recent decades, Middle Eastern literature has lagged, despite a growing prevalence of mental health needs in the region. In the US, numbers have revealed that almost one-third of people with diagnosable mental health conditions do not obtain treatment, largely underutilizing healthcare services [1]. This disparity is even more pronounced in countries of the Middle East [2].

According to the University of Chicago’s Center for Middle Eastern Studies, the Middle East consists of 16 countries: Bahrain, Egypt, Iran, Iraq, Israel, Jordan, Kuwait, Lebanon, Oman, Palestine, Qatar, Saudi Arabia, Syria, Turkey, United Arab Emirates and Yemen [3]. With rising socio-geo-political issues in many of these countries, more research exploring the region’s mental ill-health and community attitudes towards help-seeking is crucial.

### Stigma, shame and secrecy

The Middle East has a fascinating history regarding their approach to mental health, having built some of the first psychiatric hospitals in the world, for example Baghdad in 705 A.D. However, several studies have demonstrated that, in present times, the Middle East is largely suffering from negative attitudes towards mental health. For example, Al-Darmaki found that individuals react with disgrace at the prospect of requiring psychological help, exemplifying the stigmas associated with receiving therapy [4]. People dealing with mental illnesses must contend with both the symptoms of their condition and the societal stigmatisation associated with it, particularly due to the view of therapy as an intrusion that is incompatible with the region’s cultural norms and values [5].

When analysing how stigma affects help-seeking, it is beneficial to differentiate between three levels of discrimination: public, structural, and self-stigma. Public stigma refers to the stigmatising attitudes toward mental illness through labelling and discrimination [6]. Structural discrimination refers to negative outcomes brought on by inequalities ingrained in societal structures, such as insufficient mental health care coverage or difficulties accessing services [7, 8]. Self-stigmatisation, however, ensues when members of a minority adopt these prevalent stigmatising notions and begin to feel inferior to and rejected by most people.

Stigma acts as a significant barrier to recovery. In order to avoid being labelled as "mentally ill" [9], evidence from high-income countries indicates that between 44% and 70% of those who would benefit from mental health treatments choose not to utilize them [10]. This treatment gap was found to be as high as 90% in developing countries. Patients avoid getting the necessary treatment due to low self-efficacy, feelings of shame, and lack of confidence in an effort to escape the accompanying stigma, and this is particularly true among Arab populations [11, 12]. These attitudes, along with other factors such as culture and religion, are therefore affiliated with hesitation to receiving mental health care [13, 14].

### The role of culture and family in the Middle East

The family is crucial in determining whether people from the Middle East seek mental health services. In Middle Eastern society, a person’s behaviour is considered an indication of how strongly their family adheres to social norms [15] and maintaining social standing is a critical aspect that should not be jeopardized by any shameful or humiliating threats. Here, family members risk being criticized for failing to teach the behaviours that are deemed culturally unacceptable [16]. Thus, in a region where collectivism is heavily valued and self-actualisation is discouraged, the family appears to be a significant influence in determining attitudes toward help-seeking [16, 17]: a decision predominantly made by the males in the family [18].

In abiding with the traditional laws governed by family norms [19, 20], preferences to confer within the family are therefore not unusual. However, due to sentiments of shame, mental illnesses are often received with disregard or complete dejection by the families. In a study conducted in Egypt, over 50% of participants stated that they would not accept someone as a family member if they were diagnosed with a mental condition [21], causing individuals to prioritize family interests over personal interests [22].

In turn, individuals’ self-view may shift to being aberrant or atypical from the remainder of society, further disheartening them to seek mental health care [23]. This can be better understood when considering Kleinman’s explanatory models of illness (1978) [24]. He argues that social context influences the way people experience mental health, where different cultural backgrounds may result in different interpretations and attributions of mental illnesses, directly influencing opinions related to help-seeking.

Research has also demonstrated that the provision of care for severe mental illnesses such as psychosis, bipolar, or depression is mostly the responsibility of females and family members, as opposed to statutory services [25], further emphasising the contribution of a sum of factors to help-seeking behaviours in the region.

### The role of religion in the Middle East

Although the Middle East hosts a variety of religions, Islam remains the region’s predominant religion. Although Islamic principles are founded upon seeking treatment when needed [26], there remains a pervasive understanding that mental illnesses are a result of lack of faith [27]. Instead, hardships are interpreted as the idea that God has his own plan for everyone [28] leading individuals to portray mental illness as a test from God that must be accepted [29].

Muslims often seek the help of religious leaders such as Sheikhs or Imams due to their influential positions within the community [30]. So, the instinctive act of seeking help from religious leaders may be overshadowing the resourceful utilization of mental healthcare services [17], particularly due to the common belief that mental illness may occur as a result of supernatural doings. Whilst the numbers are unclear for Middle Eastern populations, a nationwide epidemiological study conducted in the United States discovered that 5% of individuals in need of mental health treatment sought it from ministers, priests, and rabbis [31]. Additionally, the stigma associated with visiting a religious leader does not match that of visiting a mental healthcare facility [32], making it crucial to consider how religious factors may be shaping help-seeking patterns among this population.

### The aims of the current research

While several systematic reviews have explored barriers to help-seeking behaviours in different regions and on specific disorders e.g. [33, 34], a recent review has investigated help-seeking experiences in Arab populations worldwide [35]. However, to date, no systematic review has specifically focused on mental health and help-seeking among people living exclusively in the Middle East. This research particularly impactful as it allows us to uncover region-specific barriers and facilitators, which may differ from those identified on a broader international scale.

Furthermore, most of the published literature on help-seeking has used Western-validated psychometric instruments and extrapolated structural, cultural, and psychosocial variables that impede help-seeking. While this has significantly contributed to our awareness of the challenges involved with mental health treatment in the Arabic world, such methodological approaches can be viewed as a setback for psychological research in cross-cultural contexts [36].

To address this gap, we conducted a systematic review synthesizing all relevant qualitative studies to explore help-seeking behaviours and mental health in the Middle East. With the research on this topic being in its infancy, this study is firmly situated in the field of qualitative enquiry as it strives to explore complex issues in a richer, more detailed manner.

The review is concerned with help-seeking from both formal (e.g., mental health services and treatment such as psychotherapy, counselling, and advice from trained professionals) and informal (e.g., advice from family, friends, or religious figures) sources. Help-seeking denotes all stages of the process linking the onset of mental health issues to accessing psychological care, covering the initiation of, engagement with care, or general attitudes towards help-seeking [37]. The research question explored in this review was: What are the behaviours that are associated with the likelihood of seeking help from mental health services in the Middle East? By investigating the facilitators and barriers that influence help-seeking patterns, this study aids in the generation of evidence-based, culturally relevant strategies to promote help-seeking in the Middle East.

## Methods

### Design

We conducted a systematic review to identify and synthesise all available qualitative data related to help-seeking behaviours and attitudes of individuals in the Middle East regarding their mental health issues. To ensure full transparency of reporting and study evaluation, we adhered to the Reporting Items for Systematic Reviews and Meta-Analyses (PRISMA) guidelines throughout the entirety of the review (See S1 File. Appendix for PRISMA checklist) [38]. We conducted a meta-synthesis to amalgamate results from published qualitative studies together, enabling us to pool data from across studies to look for deeper insight across participants and contexts that may not be available in a single study [39]. A protocol for the systematic review was registered with PROSPERO (Ref: CRD42022311494).

### Search strategy

We conducted the final systematic search of qualitative studies on the 11^th^ of July 2022 across five electronic bibliographic databases: MEDLINE, Embase, CINAHL, PsycINFO and Qscience, following search-strategy recommendations specific to each database [40–42]. To identify additional published papers, such as theses and dissertations, we also searched grey literature using Google Scholar, OpenGrey, and ProQuest Dissertations & Theses Global. We also conducted ‘hand-searching’ through screening reference lists of relevant studies. Citation tracking of these studies was used in Google Scholar to identify further papers. Backwards and forwards citation checking was completed for all included papers to ensure no papers were either missed during indexing or had not been picked up by database searches.

To capture the complexity of help-seeking among individuals in the Middle East, key search terms informing the topic were first piloted and refined using the ‘PICOS’ framework, forming three clusters of keywords: “help-seeking”, “mental illness”, and “Middle East”. Each search term was further expanded to include all possible alternative terms relevant to each database and combined using Boolean operators (see S2 File. Appendix for full search terms). We consulted with the university librarian and experts within the field before the finalization of results to ensure the search strategy retrieved maximal results.

### Selection criteria

Pre-defined inclusion and exclusion criteria were specified and documented to be used to inform our search. The inclusion criteria were that studies had to (1) employ a qualitative research design (mixed method studies had to have a qualitative component which could be extracted and analysed separately); (2) be on any type of mental health issues (depression, anxiety, bipolar disorder, schizophrenia etc.), with no restrictions placed on the type of mental illness examined; (3) be published journal articles, theses, or dissertations; (4) be conducted on Middle Eastern participants as defined by the Centre for Middle Eastern countries [3], living in the Middle East; (5) consist of a sample aged 18–65; (6) be from their earliest records (inception) up to July 2022 to ensure the inclusion of the most up-to-date and comprehensive literature available within that timeframe; (7) be written in the English or Arabic language; and (8) address either formal or informal help-seeking behaviours related to mental health, or barriers or facilitators of psychological help-seeking.

Studies were excluded if they were (1) quantitative studies; (2) unpublished studies (e.g., case studies, conference abstracts, studies reported in book chapters, editorials or letters); (3) conducted on participants aged below 18, or over 65; (4) conducted on participants from the Middle East countries in a country outside of the Middle East region; (5) conducted in other languages; and (6) on help-seeking behaviours for physical/developmental illnesses (cancer, diabetes, or autism), career or vocational problems (e.g. career choice), unless specified that there is a diagnosed/undiagnosed mental health concerns (e.g. anxiety, depression).

### Data screening and extraction

We downloaded search results and imported findings into EndNote reference management tool for deduplication and sorting. The primary reviewer FE screened all titles and abstracts and the second reviewer, AH, independently reviewed 20% of all papers retrieved at this stage to assess relevance in line with the eligibility criteria. Any topics outside the scope of Middle East participants and help-seeking behaviour for mental health issues were rejected, as well as those addressing the topic but failing to abide by the eligibility criteria. Then, full-text papers of potentially eligible studies were independently screened by both the primary and secondary reviewers. Any discrepancies were resolved through discussion with the rest of the research team. Data was then inputted in a pre-designed data extraction table comprising relevant information (See Table 1).

**Table 1:** Characteristics of included studies.

Study No.	Author(s) (Year)	Study country	Sample size	Method	Analysis	Aims
1	Al Laham et al. (2020) [55]	Lebanon	N = 46 Syrian refugees and members of local Lebanese community)	Semi-structured interviews, focus-group discussions	Thematic analysis	To explore factors, including socio-cultural beliefs, that influence mental help-seeking behaviours
2	Al-Darmaki et al. (2016) [56]	United Arab Emirates	N = 70 female college students from the full spectrum of majors (e.g., psychology, public relations, finance)	Open-ended questionnaire	Thematic analysis	To investigate Emirati females’ perceptions in relation to mental health beliefs and barriers to help-seeking
3	Al-Dousari & Prior (2020) [57]	Kuwait	N = 3 Kuwaiti counselling clients	Semi-structured interviews	Thematic analysis	To explore how Islamic faith facilitated mental health help-seeking in individuals accessing counselling
4	Alfayumi et al. (2019) [58]	Israel	N = 75 females who experienced pregnancy and childbirth	Focus-group discussions	Thematic analysis	To identify barriers to postpartum depression treatment among Bedouin women
5	Al-Kurdi (2015) [54]	Turkey	N = 5 Syrian male mental health professionals working with Syrian refugees in Turkey (3 psychiatrists, 2 psychologists)	E-mail interviews	Thematic analysis	To investigate attitudes towards mental illness and help-seeking, identifying alternative sources of help from a Syrian cultural perspective
6	Ayalon et al. (2015) [59]	Israel	N = 13 primary care physicians and 32 primary care patients with or without diagnosis of mental illness	Focus-group discussions	Thematic analysis	To examine the attitudes concerning informal help-seeking patterns to better understand mental health service use
7	Bawadi et al. (2022) [60]	Jordan	N = 24 Syrian refugees (16 clients and 8 community leaders)	Semi-structured interviews	Thematic analysis	To explore the perspectives from the host community and community leaders on barriers and facilitators to mental health service utilization by Syrian refugees
8	Hasan & Musleh (2017) [61]	Jordan	N = 27 family members of patients diagnosed with psychosis	Semi-structured interviews	Thematic analysis	To explore the factors that delay help-seeking from the perspective of the family of a patient with first episode of psychosis
9	Khatib & Abo-Rass (2021) [62]	Israel	N = 28 university students	Semi-structured interviews	Thematic analysis	To examine the mental health literacy of Arab students in Israel
10	McKell et al. (2017) [63]	Jordan	N = 16 healthcare professionals working at health centres for Palestine refugees in Jordan	Semi-structured interviews and focus group	Thematic analysis	To identify the barriers to accessing mental health services for Palestine refugees with psychological difficulties who reside in a refugee camp in Jordan
11	Nasir & Al-Qutob (2005) [52]	Jordan	N = 50 primary healthcare providers	Focus-group discussions	Thematic analysis	To explore the barriers to the diagnosis and treatment of depression in Jordan
12	Noorwali et al. (2022) [64]	Saudi Arabia	N = 12 participants from the general public	Semi-structured interviews	Thematic analysis	To explore the barriers and facilitators of mental health help-seeking among young adults in Saudi Arabia
13	Noubani et al. (2020) [65]	Lebanon	N = 36 Syrian refugees and Lebanese host community members (including caretakers of those affected by mental health issues)	Semi-structured interviews followed by participatory group model building	Thematic analysis	To determine how Lebanese host and Syrian refugee communities perceive mental health, and identify barriers to help-seeking
14	Scull et al. (2014) [66]	Kuwait	N = 10 participants (4 with a history of seeking mental health services)	Semi-structured interviews	Grounded theory	To understand the Kuwaiti perception of mental health treatment and care and identify barriers to mental health seeking
15	Taghva et al. (2017) [67]	Iran	N = 14 stakeholders of mental health (e.g., physicians, patients, family members)	16 individual interviews, 2 focus-group discussions, and 6 written narratives	Content analysis	To investigate stigma reduction barriers towards mental disorders in Iran
16	Topkaya (2015) [53]	Turkey	N = 10 participants	Semi-structured interviews	Thematic analysis	To determine which factors play a role in in adults’ decisions to seek psychological help

### Meta-synthesis

We used a meta-synthesis approach to synthesise findings from qualitative studies [43]. From all included studies, we extracted data about mental health and help-seeking behaviours in the Middle East from the results section or general text (in the case of reflective commentaries or insight being provided) and entered texts verbatim into NVivo V12 software for qualitative thematic analysis [44], a method commonly used for identifying and categorizing patterns across qualitative data [45].

First, we read and re-read each paper through the process of active reading to fully immerse in the data, creating memos for each study’s findings through coding freely. Initially, we coded results from five papers and discussed this in research team meetings, and then created themes from chunks of text for each individual study. Next, an initial coding frame was developed from each segment of data to eventually be grouped into a hierarchical structure.

Once initial codes were established, similarities and differences between them were compared and nodes were created serving as visual containers of themes. FE then developed a set of refined descriptive themes, ensuring each key theme captured similar themes across a range of papers. Themes from each article were synthesised, forming a higher-order set of themes and sub-themes relating to the relationship between mental health and help-seeking behaviours of individuals within the Middle East, creating the results of the meta-synthesis developed in the analysis. All authors collaboratively discussed the themes as a means of enhancing validity, remaining transparent about their epistemological and ontological standpoints in conducting the research.

### Quality appraisal

As recommended by the Cochrane Collaboration [46], the Critical Appraisal Skills Programme (CASP) was chosen to assess the quality of the studies due to its reputation for addressing all the necessary principles that underpin qualitative research [47].

The CASP tool was used to critically appraise the quality of studies to be included in the systematic review. Its checklist comprises guidance on appraising the methodological quality (e.g., appropriateness of recruitment strategy, design, data collection) of studies, the validity (e.g., relationship between researcher and participants) and the generalisability (e.g., consideration of ethics, rigorousness of analysis) of the results. Following Lachal et al.’s (2017) guidance on qualitative synthesis within psychiatry, studies were then weighted in 3 levels, either to have (a) totally met; (b) partially met; or (c) not meet the quality criteria [44].

### Reflexivity

As a key measure of quality in qualitative research, reflexivity is used by researchers to inwardly examine their own assumptions and consider how this may influence data interpretation [48]. The research members of this review were made up of a multidisciplinary team from Arabic and also Western points of view. This dual cultural identity led us to recognise how cross-cultural understandings of humans can be shaped based on contextual setting. We were interested in trying to understand how cultural context may shape community attitudes around mental illness, and the role it plays in psychological help-seeking.

We comprised a multidisciplinary team with varying levels of expertise on mental illness, Middle Eastern studies, and qualitative meta-synthesis. With most of the researchers originating from different countries in the Middle East (Egypt, Lebanon, and Qatar), we brought an insider’s knowledge of Middle Eastern cultural practices and values. With the supervision of a White British researcher, we aimed to limit any blind spots around the data by combining a diverse range of interpretations and questioning our presumptions of the research. The rigour and quality of this review was facilitated by continual critical reflexive discussions with the research team.

## Results

From the electronic database search, we retrieved a total of 4392 studies. Duplicate publications were removed, and the remaining 3228 papers’ titles and abstracts were screened for relevance. A total of 3199 studies were excluded at this stage, resulting in 29 papers that were fully reviewed. We excluded 15 papers for not meeting the specified inclusion criteria. A qualitative study conducted in an outpatient mental health clinic in Qatar was excluded because the authors failed to specify the nationalities of their participants [49]. Since Qatar hosts a multitude of nationalities comprising 88.4% of non-Qatari immigrants, it would be unjust to assume that all the participants included in the study were from the Middle East [50]. Similarly, a study conducted on psychological help-seeking attitudes in a Turkish university was removed [51]; having been conducted in Istanbul, Turkey’s most urbanized city, we have chosen not to include this study due to the chance that students from neighbouring countries may have sought opportunities to study abroad. This indicated that the sample may not strictly be made up of Arab participants.

**Fig 1:**
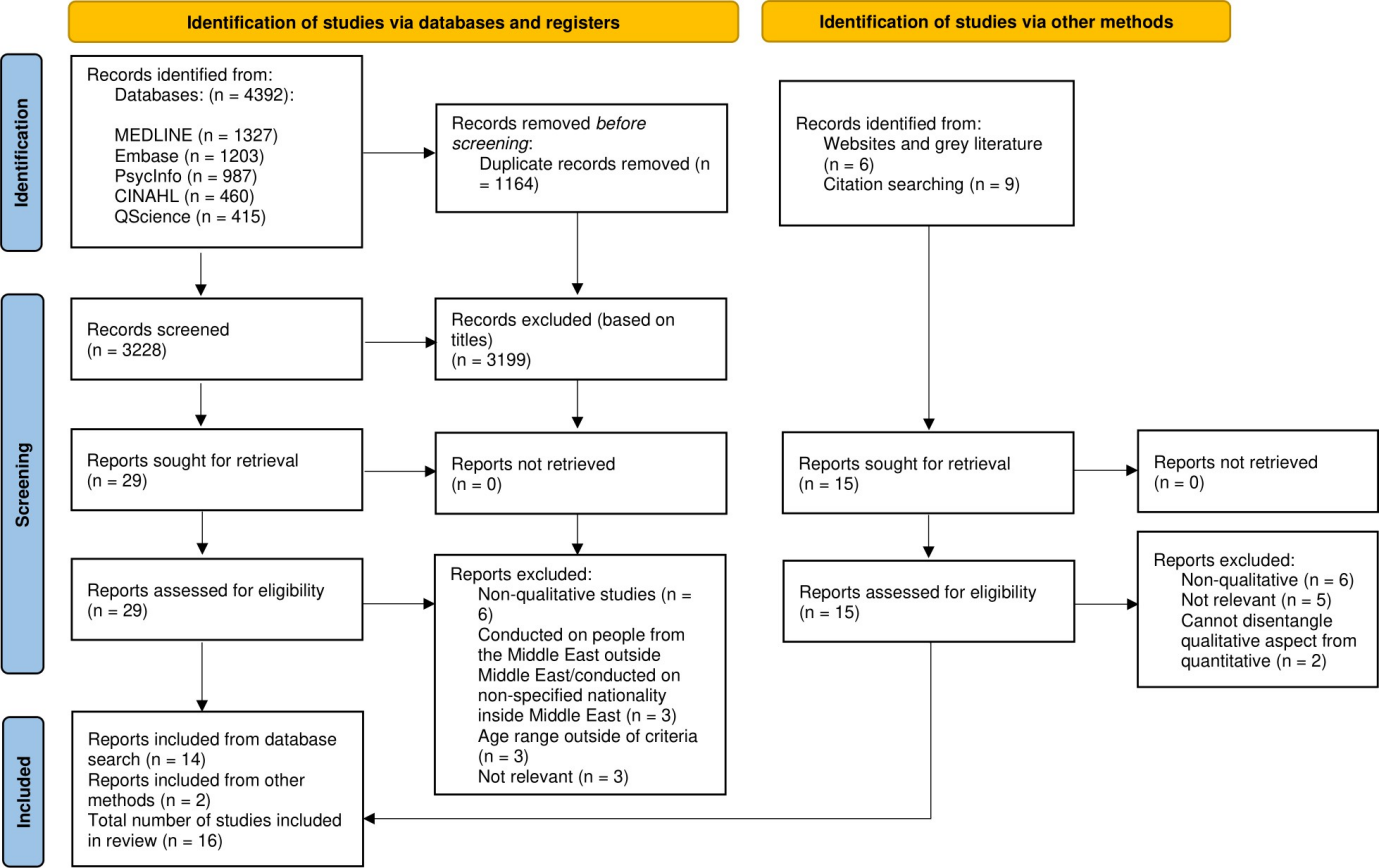
PRISMA flowchart-study selection process.

We retrieved 14 studies from database searching that were included in the review. A further nine studies were identified from the grey literature search, and six from hand-searching reference lists, of which two met the eligibility criteria. Therefore, a total of 16 studies were included in the systematic review and meta-synthesis. See (Fig 1) for PRISMA flowchart.

### Characteristics of the included studies

The studies identified were carried out in Jordan (*n* = 4), Lebanon (*n* = 3), Israel (*n* = 3), Kuwait (*n* = 2), United Arab Emirates (*n* = 2), Turkey (*n* = 2), Iran (*n* = 1) and Saudi Arabia (*n* = 1), with sample sizes ranging from 3 to 75. All studies were purely qualitative, mostly utilizing focus-group discussions or semi-structured interviews to collect data on psychological help-seeking of participants. One study used email interviews, and another used open-end questionnaires.

Seven studies explored the perspectives of stakeholders of mental health (e.g., physicians, policymakers, midwives), two of family members/caregivers of those affected by mental health issues and the remaining seven studies from a first-person point of view of the person experiencing mental health difficulties. All studies, but two [52, 53], were peer-reviewed and published in academic journals between 2005 and 2022. One study was a PhD thesis [54]. See Table 1 for study characteristics.

### Quality appraisal

Using the CASP checklist, we assessed the quality of all the included studies and decided not to exclude any studies based on quality due to the limited available research on this topic. Quality ratings for each study are shown in Table 2.

**Table 2:** Full CASP assessment on sixteen studies.

CASP questions	Study numbers															
	1	2	3	4	5	6	7	8	9	10	11	12	13	14	15	16
	Al Laham et al. (2020) [55]	Al-Darmaki et al. (2016) [56]	Al-Dousari & Prior (2020) [57]	Alfayumi-Zeadna et al. (2019) [58]	Al-Kurdi (2015) [54]	Ayalon et al. (2015) [59]	Bawadi et al. (2022) [60]	Hasan & Musleh (2017) [61]	Khatib & Abo-Rass (2021) [62]	McKell et al. (2017) [63]	Nasir & Al-Qutob (2005) [52]	Noorwali et al. (2022) [64]	Noubani et al. (2020) [65]	Scull et al. (2014) [66]	Taghva et al. (2017) [67]	Topkaya (2015) [53]
**Was there a clear statement of the aims of the research?**	T	T	T	T	T	T	T	T	T	T	N	T	T	T	T	T
**Is a qualitative methodology appropriate?**	T	T	T	T	T	T	T	T	T	T	T	P	T	T	T	T
**Was the research design appropriate to address the aims of the research?**	T	P	T	T	T	T	P	T	P	T	N	T	T	T	P	P
**Was the recruitment strategy appropriate to the aims of the research?**	T	P	T	T	T	P	T	T	P	T	T	T	T	T	P	T
**Were the data collected in a way that addressed the research itself?**	T	P	P	T	T	T	P	T	T	T	T	T	T	T	T	P
**Has the relationship between researcher and participants been adequately considered?**	P	P	N	N	T	N	N	T	N	T	N	N	P	T	N	P
**Have ethical issues been taken into consideration?**	T	T	T	T	T	P	T	T	T	T	P	T	P	P	P	P
**Was the data analysis sufficiently rigorous?**	T	P	N	T	T	P	N	T	P	T	P	T	T	T	P	P
**Is there a clear statement of findings?**	T	T	T	T	T	P	P	T	T	T	T	T	T	T	T	T
**How valuable is the research?**	T	T	P	T	T	P	P	T	P	T	T	T	P	T	T	T

T = Totally met, P = Partially met, N = Not met

### Themes

**Fig 2:**
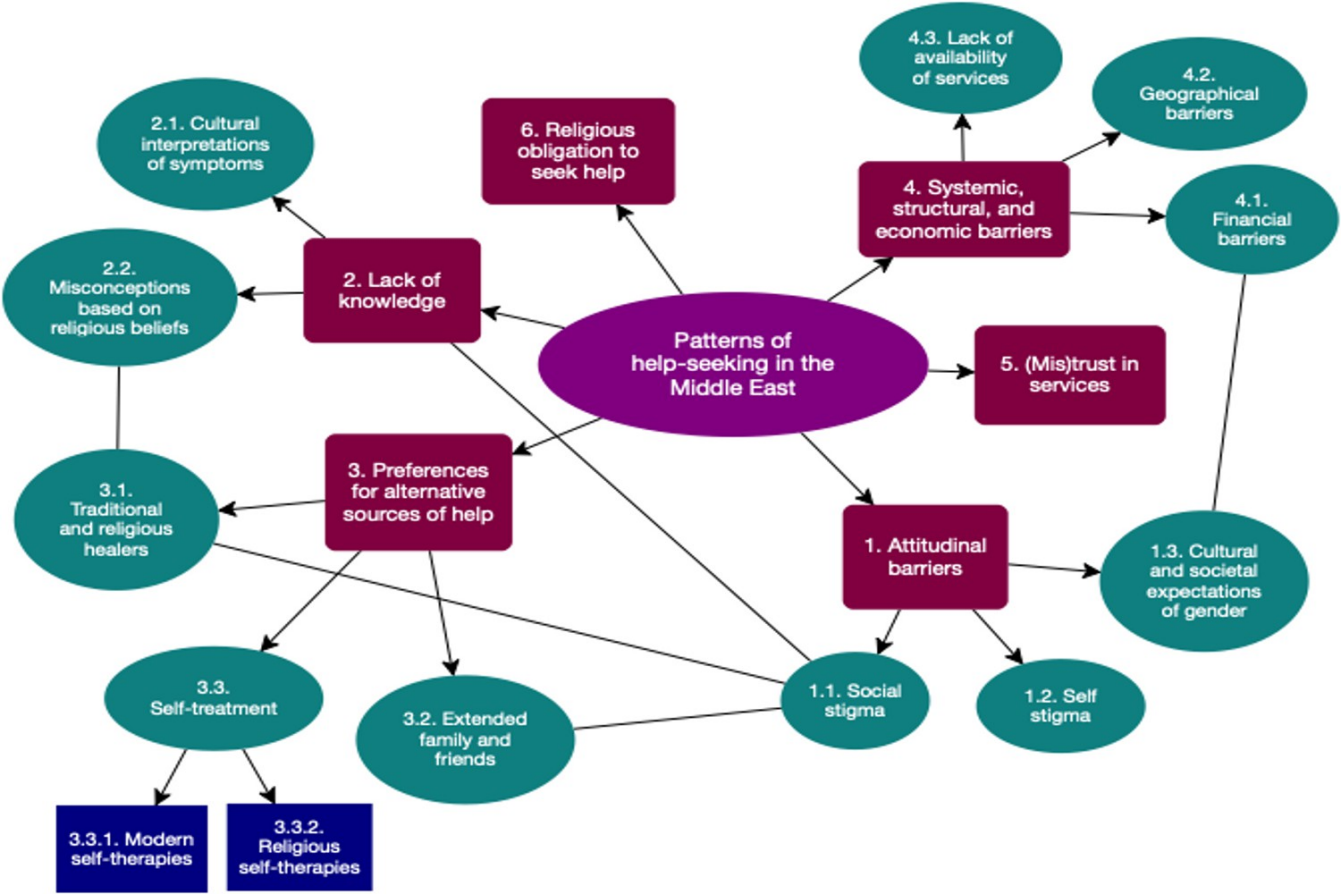
Overarching themes and associations between themes of the patterns of help-seeking in the Middle East.

From our analysis of the qualitative data, we identified six main themes related to help-seeking patterns in the Middle East (see Table 3). Corresponding to each theme, direct quotes were extracted from the included studies and presented below (see S3 File. Appendix for supplementary quotations). (Fig 2) illustrates a visual depiction of the relationships found between themes, presented using one-way and associative connectors to illustrate their interconnected nature.

**Table 3:** Overarching themes emerging from the meta-synthesis.

Key themes	Sub-themes	Sub-sub themes
1. Attitudinal barriers	1.1. Social stigma	
	1.2. Self-stigma	
	1.3. Cultural and societal expectations of gender	
2. Lack of knowledge	2.1. Cultural interpretations of symptoms	
	2.2. Misconceptions based on religious beliefs	
3. Preferences for alternative sources of help-seeking	3.1. Traditional and religious healers	
	3.2. Extended family and friends	
	3.3. Self-treatment	3.3.1. Modern self-therapies
		3.3.2. Religious self-therapies
4. Systemic, structural, and economic barriers	4.1. Financial barriers	
	4.2. Geographical barriers	
	4.3. Lack of availability of services	
5. (Mis)trust in services	-	
6. Religious obligation to seek help	-	

#### 1. Attitudinal barriers

This theme is concerned with people’s deep-rooted attitudes surrounding mental health within the region. Within this theme, social, personal, and cultural factors of help-seeking were identified, and these include stoic beliefs, societal expectations of gender roles, and self- or public stigma. As a consequence of these attitudes, many were reluctant to seek help for their mental illnesses due to feelings of fear and shame.

*1*.*1*. *Social stigma*. There was a consensus across the studies that the reluctance of individuals in the Middle East to seek help for their mental illness was out of fear of fuelling negative judgements from the community. The word ‘*majnoon’* in Arabic was used to describe those with mental illnesses across several studies. This term, whilst directly translating to *‘crazy’*, carries with it other negative connotations like ‘mad’ or ‘possessed’.

Instead of viewing mental illness as a condition that requires treatment, the community views it as a failure on the individual’s part to integrate into society, resulting in many families becoming socially marginalized from societies. Such views force individuals to hide their mental illness to avoid disgracing themselves and their families. For example, a community leader reported:

*“The community is looking at you as if you are crazy or something is wrong with you–… and people distance themselves from you*. *So*, *to avoid this talking*, *you do not go to the psychiatrist”* [60].

Similarly, some families believed that the social ramifications of getting caught seeking treatment are highly damaging to their reputations. People from the Middle East often do not look favourably upon therapy, making it difficult for patients to seek the necessary treatment. For example, a healthcare professional noted:

*“They think if they seek this kind of assistance they will be…disqualified totally from social acceptance*… *And his daughter and son will suffer*. *They think psychological disorders are genetic”* [63].

*1*.*2*. *Self-stigma*. Self-stigma was also found to be prevalent in a few studies, where participants deemed themselves socially unacceptable if they sought psychological help. As a result, they refrained from seeking services to maintain a positive image of themselves. A mental health professional described encountering many patients who prefer not to talk about their illness out of feeling *“guilty or ashamed”* [54].

As a result of self-stigma, interpersonal dilemma was observed as a factor that prevented individuals from help-seeking. This arises when there is a conflict between a person’s needs and their thoughts. A healthcare provider mentioned:

*“[a patient] feels so much shame that he waits until he is very uncomfortable*, *until he can’t stand it [to seek help]”* [52].

*1*.*3*. *Cultural and societal expectations of genders*. Across the studies, participants voiced that gender relations were often shaped by patriarchal culture with males holding the decision-making power within the family. Some female interviewees expressed that it was considered unacceptable for them to work or even leave the house without the approval of their husbands or fathers. With complete economic dependency on the males of the household, women hold minimal autonomy to seek professional help for themselves, limiting their access to necessary treatment.

Due to some societies’ perception of mental illness, women disclosing their psychological issues was a cause of great source of embarrassment to men. This often resulted in the husbands divorcing them or marrying another, making her the ‘lesser’ wife. This justified the female reluctance in seeking help, as explained by a mental health professional:

*“We have many women patients… who worry what their husband will say or think…they feel uncomfortable…we have cases where we have to sit with the male family member to give updates on the treatment sessions…this causes a great worry”* [54].

While women risked being abandoned for expressing emotional difficulties, this was not the case for the males. Instead, women were expected to remain by their husband’s side, enduring the repercussions associated with mental illness. The excerpt below elucidated this:

*“… As I am his wife and in our culture we should tolerate their behaviour … When I went to his family complaining [about] him they had asked me to wait because he is nervous person …”* [61].

Regardless, men endure societal pressures pertaining to gender roles of their own. A practitioner commented that overall, “*women seek more psychological help treatment than men”* because men are forced to focus on their *“practical male roles”* [54].

Therefore, it seems that the overall trend was that expectations relating to gender stereotypes for both men and women in Middle Eastern societies acted as a barrier to mental health-help-seeking.

#### 2. Lack of knowledge

Relating to the theme of stigma was a lack of knowledge and awareness among communities. Having limited knowledge of mental health led to the attribution of symptoms to somatic illness which prevented individuals from recognizing the signs of mental illness. This was particularly true when those surrounding them also displayed low levels of mental health literacy, as summarized below:

*“I did not think I had a psychological problem*… *My brothers were saying to me that you are young…you need to go outside and see people*… *but neither I nor they know that I have depression and my case requires treatment”* [60].

This suggests that little awareness about the nature of psychological disorders was a significant barrier to care. Moreover, many participants had little knowledge of the different mental health fields, and specifically who to reach out to in times of need. For example, a participant explained:

*“it could have been more useful knowing that psychologist services may help people*” [53].

*2*.*1*. *Cultural interpretations of symptoms*. Limited knowledge of mental illness also resulted in the cultural interpretation of symptoms. Patients often dismissed symptoms of depression and anxiety to “*feeling down and lazy”* or “*being over-worried*” [60]. In a study of five practitioners providing mental healthcare to Syrian refugees, one explained:

*“It is common and normal for people to say they have physical symptoms like heartache*, *heavy chest or like feelings of numbness in limbs*. *Usually*, *they do not come to seek help and say I am depressed*. *They don’t want to seem weak while everyone else is also suffering from similar problems…”* [54].

Arabic idioms of distress often conflate somatic and mental illness symptoms, leading to the frequent association of distress with physical symptoms. Such attributions contributed to the normalization of symptoms of distress, resulting in patients not seeking treatment for their illnesses.

*2*.*2*. *Misconceptions based on religious beliefs*. The lack of awareness surrounding mental health combined with strong religious doctrines in the region drove people to attribute their symptoms to a lack of religious belief, or *“weak faith and disobeying [God’s] commandments*” [62]. As a result, they felt that enduring hardships and strengthening their relationship with God were more appropriate than seeking professional help.

Commonly, many participants attributed their mental illness to supernatural agencies, leading to the perception of mental illnesses as possession by evil spirits, like Jinn, rather than treatable psychological conditions. For example, a Syrian woman clarified:

*“[S]ome people get nervous and have seizures*. *They say she is touched by Jinn*, *so they take her to the Sheikh*. *No one thinks that she might be sick and need a doctor*…*”* [55].

When such beliefs govern their way of thinking, patients sought alternative sources of help, such as religious or traditional healers. Another reason for this occurrence was due to the social acceptability of consulting such healers compared to a mental health practitioner. A patient with depression described:

*“the solution is to go to a “Sheikh” to read the Quran and use “ruqyah”(a healing method that involves the recitation of verses from the holy Quran…)”* [64].

#### 3. Preferences for alternative sources of help

In the region, the high stigma around accessing mental health services led individuals to seek alternative sources of help, ranging from getting advice from friends or family, reaching out to religious or traditional healers, or opting for self-help techniques.

*3*.*1*. *Traditional and religious healers*. In the Middle East, the understanding of mental illness is often rooted in religious and cultural contexts, so it is not uncommon that individuals sought methods of treatment in these contexts. With religious healers being more culturally acceptable than mental health providers, people felt safe knowing that others may assume they are seeking religious counsel rather than treatment.

In many cases, traditional healers played a role in preventing patients from getting professional treatment:

*“…We went to see a magician and he said that he expelled their souls out of my mind … He told us that there was no need to visit mental health service as medication damages body…”* [61].

*3*.*2*. *Extended family and friends*. In several studies, people suffering from mental health difficulties considered those closest to them as the frontline sources of for support. Reasons for this mainly pointed towards a combination of maintaining family reputation and following the region’s deep-rooted collectivist culture. In a study conducted in Turkey, a participant explained:

*“…In our culture*, *families are raised in a clan-like model*. *According to the moral laws of these clans*, *there are some strict rules about keeping family issues within the family*. *Therefore*, *people always try to solve family problems among themselves”* [53].

The absence of familial support was common across studies and played a key role in decisions to access treatment, particularly because consent to seek treatment often must be granted by all family members. A male with a severe mental illness revealed how his father prevented his pursual of treatment:

*“I wanted to visit a doctor a long time ago*, *but my father never allowed me to do so*. *Now my father is fed up as my behaviour is getting worse*, *so now I am allowed to [do so]”* [61].

This exemplifies the influence that external sources of support may have on individuals with mental health issues, as well as how they may be the preferred source of help by people from the Middle East.

*3*.*3*. *Self-treatment*. Several studies found patients to rely on themselves to get better, choosing between seeking modern or religious self-therapies. A minority of the studies mentioned that patients chose not to disclose their illnesses to avoid burdening their loved ones. For example, a Turkish participant confirmed:

*“At first*, *you need to beat yourself all by yourself*. *I mean you can deal with these problems without anyone’s help*. *Someone else can’t solve your problems for you”* [53].

***3*.*3*.*1 Modern self-therapies*.** Some participants noted engaging in modern self-therapies to break the cycle of suffering. These include a range of self-management mechanisms such as *“self-calming exercises like meditation”*, *engaging in sports”*, *or “occupying [my]self with house chores”* [60, 62].

***3*.*3*.*2*. *Religious self-therapies*.** More traditional self-therapies such as prayer and reading the Quran were also identified as sources of comfort by the participants. Although these practices were also coded under religious beliefs, they easily fell under the self-help theme due to the practices being regarded as a solar activity. In a study of ten Kuwaiti participants, one mentioned:

*“Islam is the solution for me*… *The first thing you think is God*… *This is what makes you calm down; just reading the Qur’an”* [66].

#### 4. Systemic, structural, and economic barriers

A common trend among studies involved difficulties in accessing treatments due to financial and organizational-level barriers.

*4*.*1 Financial barriers*. For many, a lack of financial resources acted as a barrier to seeking psychological treatments. These concerns were mainly focused on high treatment costs, minimal insurance policies, and costs of transportation. The added financial burden of seeking help led service users to seek alternative sources of help, skip appointments, or avoid treatment-seeking. A Bedouin woman seeking treatment for post-partum depression (PPD) stated:

*“A poor financial situation is a barrier to turning to a professional*, *and especially to a psychologist outside of the public health framework*. *Today*, *such visits cost a lot of money*, *and most people cannot pay a few hundred shekels for a session which we do not have”* [58].

Given the long-term chronic nature of mental illnesses, ongoing follow-up expenses and processes required for treatment, and travelling to and from appointments, acted as a large deterrent to the continuation of care, summed in the below excerpts:

*“…*. *Many cannot afford to come back for follow-up appointments for mental health*, *so they give up*. *Mental health needs more follow-up*, *more visits”* [63].

*“My problem is with car rental*, *the transport cost is 2 dinars (3 US dollars) and this amount of money is not available to me”* [60].

*4*.*2*. *Geographical barriers*. With many of the participants residing in rural areas, mobility limitation was commonly identified across studies. Factoring in high transportation costs and long physical distances, geographical inaccessibility was found to hinder treatment-seeking. For example, a student reported:

*“You need to take three buses to get to the mental health center in a Jewish city*, *where they do not speak Arabic [*.* *.* *.*] this prevents people from receiving treatment”* [62].

Certain living conditions also predicted likelihood to seek help. Those living in unrecognized villages with minimal government services and basic infrastructure found it difficult to access treatment, as demonstrated in this excerpt:

*“There is no electricity; there are no streets or transportation*. *The situation is extremely difficult and there are no services that can support women who suffer from PPD*, *such as*, *mental health clinic in the residential area*, *or public transportation to more distant clinics”* [58].

*4*.*3*. *Lack of availability of services*. A lack of adequate services being available to patients was also commonly reported across studies. Participants felt that there were no specific services available for them to consult with when feeling emotionally distressed. In one study, a college student described:

*“In my village we do not have mental health services…nor do I know any psychologist or psychiatrist in the village”* [62].

With a limited number of mental health professionals in the region, large demands were placed on those practising. With practitioners being overworked, they were often forced to provide inadequate care. This deterred users from seeking help:

*“There are insufficient services in our city because of the huge demand*. *Therefore*, *people avoid getting help*. *But I think if there were sufficient facilities*, *everyone would go”* [53].

#### 5. (Mis)trust in services

The theme of trust was prevalent in several studies. Syrian refugees commonly reported feeling that healthcare providers did not understand their cultural backgrounds, which decreased their help-seeking tendencies. A practitioner for Syrian refugees explained:

*“Syrians are coming across mental health care that is dominated by Western practices… and they feel this discredits their cultural or religious or social practices or values they hold…it becomes a choice of values for them than a source of help”* [54].

However, this was contradicted by a Kuwaiti participant who suggested that help would more likely be sought if their therapist was foreign-born, describing feeling more *“secure than with the Kuwaiti therapist”* [66].

The lack of trust in the overall system to improve patients’ conditions also extended to a lack of trust in the services being provided, which intensified avoidance of treatment-seeking. For instance, a Kuwaiti participant described:

*“It’s for crazy people*… *There is a [high] percentage I mean—they think that this place [hospitals and clinics] in Kuwait you cannot trust*. *You cannot*… *If you go there you will become more crazy”* [66].

Across many studies, mental healthcare was described as lacking professionalism, training, and quality of care. For example, a recurring concern were breaches in confidentiality: patients were afraid to seek help from professionals because they believed their clinicians were not trustworthy, as exhibited in the excerpt below:

*“[My psychiatrist] called one of his patients…I think she was deeply manic or she was having an episode of some sort*. *She [interrupted] our session [and] he told me*, *“don’t worry*, *that’s one of my patients*, *but you’re not like her*, *she’s crazy*.*” Quote*… *That is exactly what he said*: *“you’re not like her*, *she’s crazy”* [66].

The negative personal experiences with mental health professionals reported by individuals were also due to symptoms being overlooked or misdiagnosed. Participants mentioned that their mental health symptoms were often misinterpreted by healthcare providers due to incompetency and a lack of training in the mental health field. A young Saudi adult explained that his condition worsened after visiting a therapist. He explained: *“I remember going out of the therapist’s session feeling extremely sad”* [64].

On the other hand, healthcare workers who successfully built a therapeutic relationship with their clients found patients to return to them after initial consultations, highlighting that establishing trust with an informed provider facilitated help-seeking. An example of this was presented by a nurse-midwife:

*“Some [patients] come to you and when you talk to them they feel safe*, *and you find they come back time and again… In certain ways*, *we speak to them*, *they get better… she comes back happy and she wants to speak more…”* [52].

#### 6. Religious obligation to seek help

Despite the lay beliefs that associate mental illness with weak faith, few participants demonstrated an understanding of the correct Islamic teachings which impose an obligation to avoid self-destructive practices and seek help when needed. They also made the distinguishment that there is no conflict between Islam and mental healthcare and that they may, instead, be mutually supportive. This indicates that religious faith acted as a facilitator for help-seeking for Middle Eastern individuals whose views followed scripture, rather than cultural norms. In a study conducted in Kuwait of three females who attended counselling for over a year, one said:

*“If you are genuinely practising the teachings of Islam then you need to take all possible means to be better*. *Our religion encourages us to ask for help”* [57].

In the same study, a different participant referenced the Hadith (the prophet Muhammad’s sayings, which serve as a primary source of guidance for Muslims beyond the Quran) to emphasise a religious duty to seek assistance in mitigating suffering. She explained:

“*You know the Hadith has interpreted that Allah has not made a disease without appointing a remedy for it…Islam calls us to make use of all available means*, *to do whatever is possible to be better in all aspects of life*, *including psychologically*.*”* [57].

Islamic perspectives clearly play a significant role in shaping daily life of the studies’ participants. This influence extends to mental health care, which emerged as a recurring theme in the review of the literature. In one of the studies, participants expressed the belief that Islam and mental health care are not at odds; instead, they can complement each other. For instance, two participants reasoned that these two aspects of life are not in conflict, emphasising that Islam consistently seeks the well-being of individuals. They argued that seeking professional help for mental health issues aligns with Islamic values. A participant explained:

*“They’re not against each other*. *They wouldn’t create a conflict because our religion*, *it’s always looks for the best for the human being*. *If they’re having any mental problem or anything that might lead them to suicide*, *that’s against our religion*. *So I think it’s for the best if someone goes to a doctor to help him solve his problems or just talk*.*”* [66].

## Discussion

This study presents an in-depth analysis and meta-synthesis of patterns of help-seeking for mental illness among a Middle Eastern population. To our knowledge, this is the first systematic review that combines all published qualitative literature conducted in the Middle East and highlights the facilitators and barriers that influence help-seeking behaviours. We included sixteen studies in the review, revealing that help-seeking patterns were impacted by: 1) people’s attitudes; 2) lack of knowledge around mental illness; 3) the different sources of help; 4) financial, systemic, and structural issues; 5) lack of trust in services and providers; and 6) whether they had the correct Islamic teachings to recognize their obligation to seek help.

Westerners have demonstrated significant progress in their knowledge and attitudes towards mental illness over time [68]. However, the results of our study found that the Middle East remains fuelled by prominent misunderstandings and negative stereotypes surrounding mental illness. This is contradictory to earlier studies which reported that mental illness is not as stigmatised in the Arab world as in other societies [69, 70]. However, such studies were published in the early 1990s meaning that they may not necessarily be representative of more recent shifts in public attitudes. According to more recent studies examined in this review, stigma against mental illness is still substantially present in the Middle East and directly impacts mental health-seeking behaviours and access to psychosocial treatment, as supported by findings from a Jordanian study reporting that 62% of female domestic abuse victims felt embarrassed at the prospect of seeking support from mental healthcare services [71]. Comparable patterns were found in Muslims in the United States, indicating that cultural and religious beliefs may be the amplifying factors of stigma within the region [72]. This underscores the enduring influence of factors such as family roles, cultural values, and religious beliefs on stigmatisation in individuals from the Middle East, regardless of their location, whether they live in the West or the East. While this phenomenon is not limited to Middle Eastern cultures and has broader cross-cultural implications, the strong emphasis on collectivism and family interconnectedness in the Middle East compared to the West, may contribute to increased stigma surrounding seeking help for mental health issues [73]. For example, seeking such help could be perceived as a personal failure that brings shame to the family. Consequently, family members often encourage individuals to rely on familial support rather than seeking external help.

Participants from the studies in this review often chose to maintain their privacy by confining privileged matters to their immediate families. A qualitative study on 35 participants from the Middle East living in Australia highlighted the significant role relatives had on decisions to seek treatment, including disallowing access to it out of fears of community gossip [29]. This reflects how self-efficacy becomes sacrificed for the sake of the family, underlining how collectivism in Middle Eastern society reinforces interdependence and prioritization of family interests [22]. This lends its support to the present review’s findings relating to the influence of gendered social roles on help-seeking behaviours, a finding consistent with wider literature [74, 75].

Our meta-synthesis concluded that, unlike Western countries, people from Middle Eastern cultures made little distinction in their expressions of physical and psychological health. This directly supports Kleinman’s explanatory model (1978) which argues that manifestations of symptoms and the way distress is expressed depend on social context and vary across cultures [24]. This was corroborated by existing literature from Arab countries finding that most individuals somatise their illness [17] and normalize mental disorders [76] to avoid labelling. This may be because, in non-Western cultures, emotional expressions often evoke unfavourable social responses whereby physical expressions foster empathy from others. This contributed to how the present review’s participants demonstrated decreased awareness of their need to seek psychological treatment, coinciding with a growing global movement to seek proof for biological causes of mental illness in order to lessen stigma [77].

As a result, this review’s participants frequently sought help from unorthodox sources rather than mental health professionals, explained by wider literature to be a result of the less stigmatised nature of such alternatives [78]. In this study, participants frequently favoured consulting religious scriptures or healers as their first point of contact for mental health issues. This generalizes to other studies conducted in Arab-Muslim countries [79], but also those outside of the Middle East. Asian families, for example, are often more likely to consult faith healers before seeking help from a psychiatric clinic [80].

Our results also made clear that people fiercely rejected the explanation that mental illness manifests as an internal upheaval of emotions due to their strongly held religious and superstitious beliefs. This forms a direct contrast with beliefs in Western cultures that attribute mental disorders to biological or psychosocial aetiologies [81]. As in Walpole et al. (2013), participants from the present study also attributed psychosocial problems to weakness of faith or supernatural schemas [27]. Thus, seeking mental healthcare from professional sources was influenced by the extent to which individuals adhered to their cultural conceptualizations of mental health issues.

Outcomes from this study provide insight into the intricacy of help-seeking behaviours, supporting earlier research findings that structural barriers hinder help-seeking in lower-income countries [82]. As most of our findings were derived from developing countries, people’s material circumstances indicated high levels of basic unmet needs, which overshadowed the priority to seek psychological intervention. These barriers were less frequent in more developed countries of the Middle East. As an example, statistics from Saudi Arabia showed that as well as providing free healthcare, the availability of mental health professionals was above the global median range [83], indicating that personal attitudes were a larger determinant of help-seeking than the accessibility of services in the resource-rich labour-importing countries (e.g. Qatar and Kuwait).

Whilst there was minimal discussion on facilitators that encouraged help-seeking, participants frequently cited that the enhancement of societal and family awareness would have motivated them to seek help for mental health concerns [55, 58]. This is connected to the idea that awareness serves as a component of education, ultimately resulting in enhanced mental health and a decrease in the prevalence of stigma. Additionally, advocating for increased accessibility of mental health services and providing free services were also recommended as measures to facilitate and encourage help-seeking in the region [53, 65].

### Strengths and limitations

This meta-synthesis contributes to existing knowledge on people from the Middle East’s conceptualisations of mental illness, exploring different barriers and facilitators to help-seeking behaviours within their cultural and social context. The use of a meta-synthesis to combine data from qualitative studies and reveal emerging overarching trends across the data allowed for both a broad and deep examination of participants’ experiences and perspectives. Another clear strength of this review was its rigorous and systematic methodology. Consultations with librarians and experts within meta-syntheses and Middle East studies facilitated a comprehensive search strategy. Additionally, independently screening and assessing the quality of the papers with another reviewer also ensured the accuracy of the included studies.

One crucial limitation of this review is the dearth of research in this area, which restricted the number of studies yielded. In addition to this, while the search was limited to English and Arabic languages, there were no studies retrieved in the latter. Searching databases that may be more widely used in the Arab world, such as *The Arab Journal of Psychiatry*, may have retrieved more results in this language. Another drawback of this review was not having access to the original data presented in the included studies. This meant that the ability to find cross-cutting themes was mainly based on what had previously been reported in the published studies.

There was also little diversity in the included studies. No results were yielded in certain countries like Egypt, Iraq, or Oman, for example, meaning that the results may not necessarily be transferable to all the countries within the Middle East. With the majority of studies discussing help-seeking barriers rather than facilitators, the provision of a more balanced review was compromised in this study.

### Implications for future research and practice

The all-pervading effects of stigma influenced many of the outlined barriers, highlighting the critical need to combat social and structural discrimination by targeting public stigma. Initiating informal networks rooted in the same social environments, such as collaborating with women’s self-help organisations or village aid may be an appropriate starting point for transformation. Valuing everyone’s right to self-determination and developing more sensitive societal standards may encourage enhanced support and compassion towards help-seeking within communities. Additionally, in order to reach communities in remote locations or with low literacy rates, psycho-educational interventions must be developed as well-designed, user-friendly, and culturally approachable modes of communication.

Collaborating with Imams and Sheikhs could greatly enhance the support that mental health professionals offer to individuals from the Middle East who are grappling with mental health challenges. A study involving 62 Imams in the U.S. revealed that 95% of them devoted a substantial amount of time to providing counselling to members of their community [84]. This underscores the pivotal role Imams play in advocating for mental well-being, given their esteemed status within the community. Armed with a deep understanding of the multifaceted issues the community faces and the intergenerational trauma it endures; they are well-equipped to address the self-stigmatisation often experienced by Muslims seeking help for mental health concerns. However, their training primarily pertains to mental health, leading them to interpret issues more through the lens of environmental factors rather than physiological elements like serotonin receptor imbalances. Collaborative efforts between Imams and mental health professionals should thus focus on mutual learning, ultimately yielding improved mental health outcomes in the Middle East. To facilitate this, Imams should first undergo training to identify mental health problems. Subsequently, they can use their trusted network to appropriately refer individuals to qualified experts, culminating in the development of both exemplary and cooperative practices that ensure comprehensive treatment plans for community members.

It may be valuable for future research to evaluate the impact of societal campaigns in increasing understanding and reducing stigma. With very little research in this area to date, future research must aim to assess the impact that demographic variables (e.g., socioeconomic status, age, setting, and culture) have on help-seeking, and examine whether integrating informal and religious support with professional care would be a more appropriate and relevant strategy for individuals in the Middle East.

## Conclusion

The findings of this systematic review and meta-synthesis highlight the facilitators and barriers that impact mental help-seeking in the Middle East. We identified a need for increased psychoeducation and community support which may help in reducing the burden of stigma on people in the region. We hope this will support mental healthcare professionals in promoting interventions that are both evidence-based and culturally informed with the goal of improving mental healthcare globally.

## Supporting information

S1 File PRISMA checklist. (PDF)

S2 File Full list of search terms. Displaying the full list of search terms that were applied across databases.
(PDF)

S3 File Supplementary table of quotes related to each theme. (PDF)
