# Supplementary material for: Mental illness and help-seeking behaviours among Middle Eastern cultures: A systematic review and meta-synthesis of qualitative data
Source: PLoS One. 2023 Oct 26;18(10):e0293525. doi: 10.1371/journal.pone.0293525 (PMC10602270; doi:10.1371/journal.pone.0293525)
Supplement: S2 File — Displaying the full list of search terms that were applied across databases. (PDF) [file pone.0293525.s002.pdf]

The database search was conducted making use of a list of predetermined search terms derived from three main keywords: “help-seeking”, “mental illness”, and “Middle East”. These were further elaborated to include alternative terms and applied across sources using the advanced search option, PICOS structure and MeSH terms combined using Boolean operators.

(“Patient acceptance of health care”) **OR** ((help or care or support or treatment) adj3 seek*) **OR** (((((Mental health adj3 (seek* or treatment*)) or attitude) adj3 (help-seek* or seek mental health servic*)) **OR** barrier) adj3 (treatment* or help-seek*)) **OR** health care utili?ation).

AND

(Mental illness) **OR** (mental or emotion* or psychological) adj3 (disorde* or illness* or problem* or health or wellbeing or distress)).

AND

(Middle East) **OR** (MENA OR Gulf Peninsula **OR** Gulf countr* **OR** Persian Gulf **OR** Arab* Peninsula **OR** Bahrain **OR** Iran **OR** Iraq **OR** Israel **OR** Jordan **OR** Kuwait **OR** Lebanon **OR** Oman **OR** Saudi Arabia **OR** Egypt **OR** Qatar **OR** Syria **OR** Turkey **OR** United Arab Emirates **OR** Yemen **OR** Palestine).
