# Supplementary material for: Mental illness and help-seeking behaviours among Middle Eastern cultures: A systematic review and meta-synthesis of qualitative data
Source: PLoS One. 2023 Oct 26;18(10):e0293525. doi: 10.1371/journal.pone.0293525 (PMC10602270; doi:10.1371/journal.pone.0293525)
Supplement: S3 File — (PDF) [file pone.0293525.s003.pdf]

Themes	Sub-themes	Respondent characteristics	Related quotes
Theme 1. Attitudinal barriers	1.1. Social stigma	Psychiatrist	*"[labelling] casts shame on the patients and their family amongst the community and ultimately affects peoples decision to seek help or adhere to prescribed treatment" (Al Kurdi, 2015)*
		College student	*"We Arabs do not look favorably upon mental therapy, think that mental therapy is not helpful, [and] identify those who seek mental therapy as a weak and crazy person [. . .]. It's [therefore] difficult to seek treatment and most who do so hide the fact that they're in therapy" (Khatib & Abo-Rass, 2021)*
		Wife of a man with severe mental disorder	*"… I felt alone when several of my friends decided to discontinue their relationship with me after my husband's aggressive period … as a result, we were being discriminated against by other people in our district and we moved home …" (Hasan & Musleh, 2017)*
	1.2. Self stigma	College students	*"refusing to confess that they have a psychological disorder or may be feeling shame" (Al Darmaki et al., 2019)*
	1.3. Cultural and societal expectations of gender	Housewife	*"My husband does not help with housework, demands that food be prepared, and the house to be clean at all times, and complains; 'You're at home all day at home and do nothing!"* **(***Alfayumi-Zeadna et al., 2019)*
		Medical professional	*"No one will marry a girl if she is known to have had a mental illness. Mental health does not affect men as much – they can still marry. So it is more of a problem for women." (McKell et al., 2017)*

Theme 2. Lack of knowledge	-	College student	*"I know that rehabilitation is provided to people after physical injuries, not for people with mental illness" (Khatib & Abo-Rass, 2021)*
	2.1. Cultural interpretations of symptoms	Psychiatrist	*"expressing mental distress in Arabic would in one sentence express bodily symptoms and state of mind…the language is made up this way maybe because definitions of illness in our culture consider both body and mind so it is hard to escape this when talking about it" (Al Kurdi, 2015)*
		Parent of a patient with a severe mental disorder	*"… Previously we thought that this illness [schizophrenia] resulted from the possession of devils inside his body which asked him to perform these behaviours. Also, we (82) thought this illness stemmed from being nervous, stressed or some unknown fear …" (Hasan & Musleh, 2017)*
		45-year-old male	*"I am always thinking so much to the point that it is causing me a migraine, and from the tension, I had stomach ache … I used to see doctors specialized in internal medicine and digestive diseases, and they were not aware of what I have … I was thinking these are symptoms of physical disease, even specialists did not know from the symptoms that it is a psychological problem." (Bawadi et al., 2022)*
	2.2. Misconceptions based on religious beliefs	College student	*"I believe that prayers and observance bring relief to mental problems, can help a person cope with his mental disorder and may even lead to healing". (Khatib & Abo-Rass, 2021)*
		19-year-old male	*"[E]ven mental health cases are due to the weakness of faith… It happens when the person starts to behave abnormally. If the person said bad things about God, he is sure touched by Jinn, because faithful men don't say such things." (Al Laham et al., 2020)*

Theme 3. Preferences for alternative sources of help	3.1. Traditional and religious healers	Primary care provider	*"before they arrive, on their first visit, they have already been to a religious figure and got some medicine to get Satan out." (Ayalon et al., 2015)*
		55-year-old woman	*"First we took him to a sheikh, he hit him on the stomach. It took him a whole year with the sheikh. He didn't feel better except with medication…." (Al Laham et al., 2020)*
	3.2. Extended family and friends	Young adult	*"When I tried to end my life, my friend thought I should seek some help to stop this feeling and move on" (Noorwali et al., 2022)*
		Person seeking help for a family member	*"My parents and grandparents were big on "problems stay within the family." They don't understand the idea of confidentiality; "that's still another person... this is Kuwait; keep the problems within the family." (Scull et al., 2014)*
		Female with post-partum depression	*"I personally knew the woman who killed her children following PPD. She had no family support. Only two days before the tragic incident, she told her family that she had suicidal thoughts, but no one took her seriously." (Alfayumi-Zeadna, 2019)*
	3.3 Self-treatment	24-year-old male	*"I stayed a long time believing that I could solve my problem and this made my life worse." (Bawadi et al., 2022)*
Theme 4. Systemic, structural and economic barriers	4.1. Financial barriers	Family member of patient with severe mental disorder	*"Antipsychotic medication is very expensive as well as lab tests… so we decided to use a less expensive method…" (*Hasan & Musleh, 2017)
		30-year-old woman	*[M]oney […] I saw doctors in Wadi Khaled and they wanted to transfer me to a psychiatrist in Tripoli, but I couldn't because of the lack of money, and they tell*

			you that the therapy will last minimum a year and that you have to buy medications every month." (Al Laham et al., 2020)
		22-year-old female	*"Difficulty of transportation, last time the weather was rainy and I do not have money for transportation so I did not come." (Bawadi et al., 2022)*
	4.2. Geographical barriers	18-year-old male	*"The centre is far away from us, today we boarded 3 buses until we arrived. Sometimes transportation is available to us and sometimes not, today we suffered a lot until we arrived." (Bawadi et al., 2022)*
		Teacher	*People are "far from the city, difficult roads and there is lack of services" (Al Laham et al, 2020).*
		23-year-old male	*"I can't get psychological services from anyone in Kütahya. But I could in Eskişehir. Of course, it is difficult for me to go Eskişehir. If we had a psychological counselor in our faculty, it would be much easier to receive psychological help because, that way, you can always find someone to talk with." (Topkaya, 2015)*
	4.3. Lack of availability of services	College student	*"It's difficult to find mental help or emotional support by phone or online in Arabic, most of it is in Hebrew. This is very difficult, especially for teenagers or other at-risk groups…" (Khatib & Abo-Rass, 2021)*
		Practitioner	*"I have too many patients in a day, often more than 80 a day. I don't have enough time to stay and talk." (McKell et al., 2017)*
		Mother of a patient with mental disorder	*"I took my son and they said they aren't treating children in this health centre... I stopped my son's treatment because there were no services for children in the city where we live and the journey was far away" (Bawadi et al., 2022)*

Theme 5. (Mis)trust in services	-	24-year-old male	*"it is necessary to trust the expert…he has to make me feel that I can trust him. First of all, what he advice is the most important thing for me to gain his trust." (Topkaya, 2015).*
	-	Lay participant	*"with a Kuwaiti [therapist] . . . there is no [security]. They are afraid that [they] will talk about them to someone they know." (Scull et al., 2014)*
	-	Patient with a history of seeking mental health services	*The problem is the hospital... They [just] test for how smart you are, but when you need to talk, they talk before you and they don't listen [to you]. It's not right what's going on in Kuwait... Actually they lost my file as well . . . They don't want to communicate with you . . . It's all bullshit, they're not helping. (Scull et al., 2014)*
	-	40-year-old male	*"I visited a doctor, but when I told him I am tired and have a headache he told me all of us feel like this. He could have said that my blood tests are normal and that I should see a psychiatrist, especially as he knows that I am Syrian." (Al Laham et al., 2020)*
	-	College student	*"He informed me about the diagnosis and it put everything into perspective; the symptoms made sense, I was able to notice them and put a label on them." (Noorwali et al., 2022)*
Theme 6. Religious obligation to seek help	-	Patient attending counselling	*"There has been a huge misconception between what teachings are of religion and what are the cultural. So, the real essence of Islam is extremely different from the way people are behaving and believing regarding help-seeking attitudes. (Al-Dousari & Prior, 2020)*
	-	Lay participant	*They're not against each other. They wouldn't create a conflict because our religion, it's always looks for the best for the human being. If they're having*

			any mental problem or anything that might lead them to suicide, that's against our religion. So I think it's for the best if someone goes to a doctor to help him solve his problems or just talk." (Scull et al., 2014)
